# Clinical outcome and explant histology after using a cellular bone allograft in two-stage total hip arthroplasty

**DOI:** 10.1186/s13018-020-1542-x

**Published:** 2020-01-16

**Authors:** Cambize Shahrdar, Julie McLean, Elena Gianulis, Davorka Softic, Xiaofei Qin, Mark A. Moore, Jingsong Chen

**Affiliations:** 1 The Orthopedic Clinic, Shreveport, LA USA; 2 LifeNet Health, Virginia Beach, VA USA

**Keywords:** Bone regeneration, Osteoblasts, Total hip arthroplasty, Allograft, Bone void filler, Osteogenic

## Abstract

**Background:**

Although use of cellular bone allografts (CBA) in orthopedic surgery has become increasingly common, little information is available regarding their short-term clinical performance. In these two case reports of two-stage hip arthroplasties, ViviGen Formable CBA (V-CBA) was used in stage one to fill voids left by previous metal implants.

**Methods:**

The two patients had distinctly different health profiles, but each of them had previous metal implants due to a hip fracture. In the otherwise healthy 49-year-old male patient, the total hip arthroplasty (THA) was performed 7 weeks after nail removal and V-CBA backfill. In the 64-year-old female patient with Type 1 diabetes and severe osteoporosis, stage 2 was performed after 12 weeks. At the time of THA for each patient, bone containing some V-CBA was removed to accommodate the hip implant. The explants were histologically analyzed for bone matrix, mineralization, and neovascularization.

**Results:**

Histological staining showed substantial new bone formation and neovascularization in both explants albeit at different levels of maturity.

**Conclusions:**

Although limited, these results suggest that V-CBA may facilitate new bone formation in healthy as well as in metabolically challenged patients.

**Level of evidence:**

V, case report

## Background

Total hip arthroplasty (THA) is a commonly performed and successful procedure [1]. However, challenges can arise when the patient has had prior hip surgery, leaving metal hardware in the proximal end of the femur or in the femoral shaft [2]. Removal of this hardware can result in substantial bone loss and stress risers, which may lead to post-operative complications following a THA [3]. In such cases, the surgeon may choose to perform a two-stage THA to restore the bone stock in the first stage using a bone graft, such as autograft or allograft. The timing of the second stage, which may occur several months later, is determined to some degree by how quickly bone consolidation occurs, which is related to the choice of bone void filler and its properties.

When choosing a bone void filler, autograft bone is the historic gold standard because it has the potential to provide all three properties necessary for bone formation: osteoconductivity, osteoinductivity, and osteogenicity [4]. However, autograft risks donor site infection, additional pain, and may be of poor quality depending upon the patient’s health [5]. Synthetics, such as bone cement, are another popular choice due to their ready availability and low cost. However, cement cannot incorporate into bone, and if a revision is needed, it risks additional loss of healthy bone during its removal [6]. A more recent category, cellular bone allografts (CBAs), presents a more physiologic alternative. CBAs contain osteoconductive bone scaffold, osteoinductive demineralized bone matrix, and viable cells that are potentially osteogenic. Most CBAs contain mesenchymal stem cells that have the potential to differentiate into bone cells, but may also differentiate into other non-bone-forming cells, such as adipocytes, tenocytes, and myocytes. Unlike other CBAs, ViviGen Formable cellular bone allograft (V-CBA) was developed to be similar to healthy autograft: it contains viable, osteogenic lineage-committed bone cells in an osteoconductive cortico-cancellous matrix along with demineralized bone with osteoinductive potential [7, 8].

Although use of CBAs in orthopedic surgery has become increasingly common, little information is available regarding their short-term clinical performance. In these case reports of two-stage THA, V-CBA was used in the first stage to fill bone voids left by previous hardware. When the patients later proceeded to THA, a small portion of the implanted V-CBA was removed and subjected to histological analysis for new bone formation. These cases presented a unique opportunity to assess the short-term clinical performance of a cellular bone allograft, V-CBA, in two patients with distinct disease profiles.

## Methods and case details

### Case #1

**Fig. 1:**
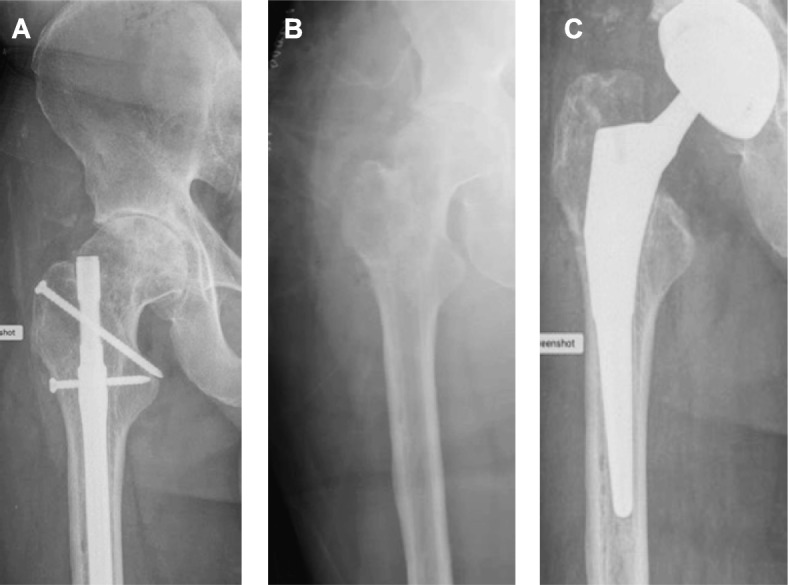
Radiographic images of patient #1 before and after THA. **a** Pre-operative radiograph showing advanced hip osteoarthritis and a femoral nail from previous fracture approximately 20 years prior. **b** Post-operative radiograph from the first procedure (Stage 1) after the femoral nail was removed and the void was filled with V-CBA to rebuild the bone stock. **c** Post-operative radiograph from second procedure (Stage 2) showing the hip replacement hardware. The second procedure was performed 7 weeks after the first procedure

**Fig. 2:**
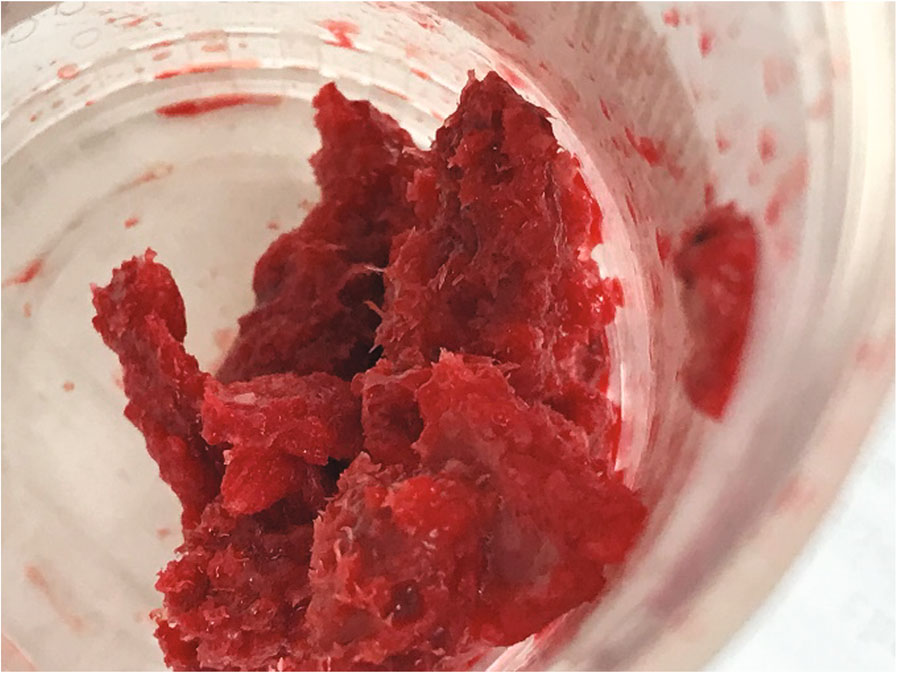
Explanted specimen. During the hip replacement (Stage 2), the femoral head and part of the neck were removed, and the explanted bone was sent out for histological analysis

Patient consent was obtained for publication of this case. A healthy 48-year-old male presented with hip pain. His Harris Hip Score (HHS) was 59 [9]. Conventional radiograph showed advanced osteoarthritis in his right hip (Fig. 1a). Approximately 20 years earlier, the patient had experienced a femur fracture, which was treated with a femoral nail. The nail’s location would interfere with the THA and needed to be removed. In this two-stage procedure, the femoral nail was removed, and the void left by the fixation hardware was filled with 10 cc of V-CBA (ViviGen Formable; LifeNet Health, Virginia Beach, VA) as shown in Fig. 1b. Radiograph showed good bone consolidation at 7 weeks, at which time the THA was performed (Fig. 1c). An anterior approach was taken using a 60-mm-diameter G7 acetabular shell, 40-mm-inner-diameter Vitamin E polyethylene insert, Taperloc femoral stem size 13 high offset, and Biolox Delta 40-mm-diameter ceramic head with a titanium alloy +3-mm taper adaptor (Zimmer Biomet, Warsaw, IN). The head of the femur and a portion of the neck, which included a small amount of the implanted V-CBA, were removed to place the total hip implant, and the explant was sent to the V-CBA manufacturer for histological analysis (Fig. 2). The patient was discharged on the same day of surgery and had no complications post-operatively.

### Case #2

**Fig. 3:**
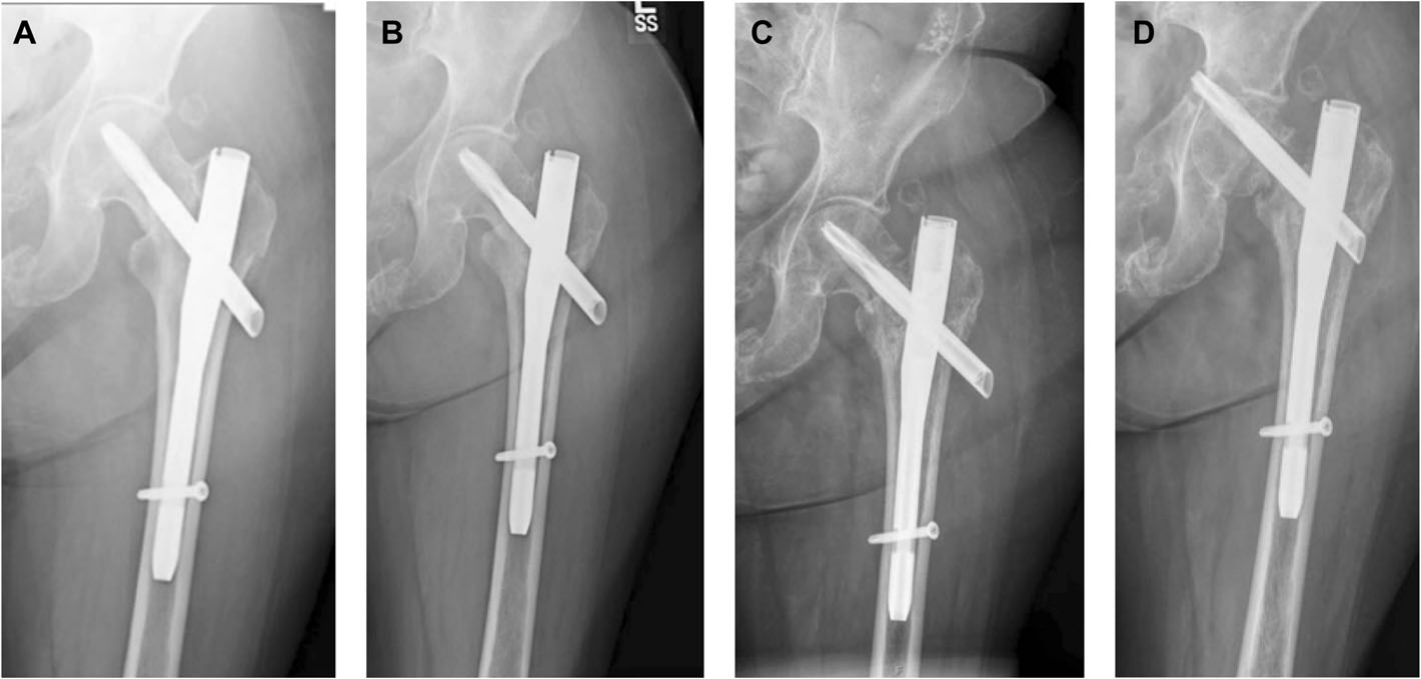
Radiographic images of patient #2 before and after THA. Radiograph at patient presentation showed Titanium Trochanteric Fixation Nail (TFN) System implanted in January 2018 after the patient fell (**a**). Radiographic images taken at 7 (**b**) and 8 (**c**) months after the fixation surgery showed that the fixation hardware had shifted and was causing severe pain. The patient experienced worsening pain from 7 to 8 months. Radiograph (**d**) 11 months after placement of the TFN System, and day 1 of Stage 1 of the THA procedure. The image shows Helical blade had perforated the patient’s femoral head and was in her pelvis and acetabulum

**Fig. 4:**
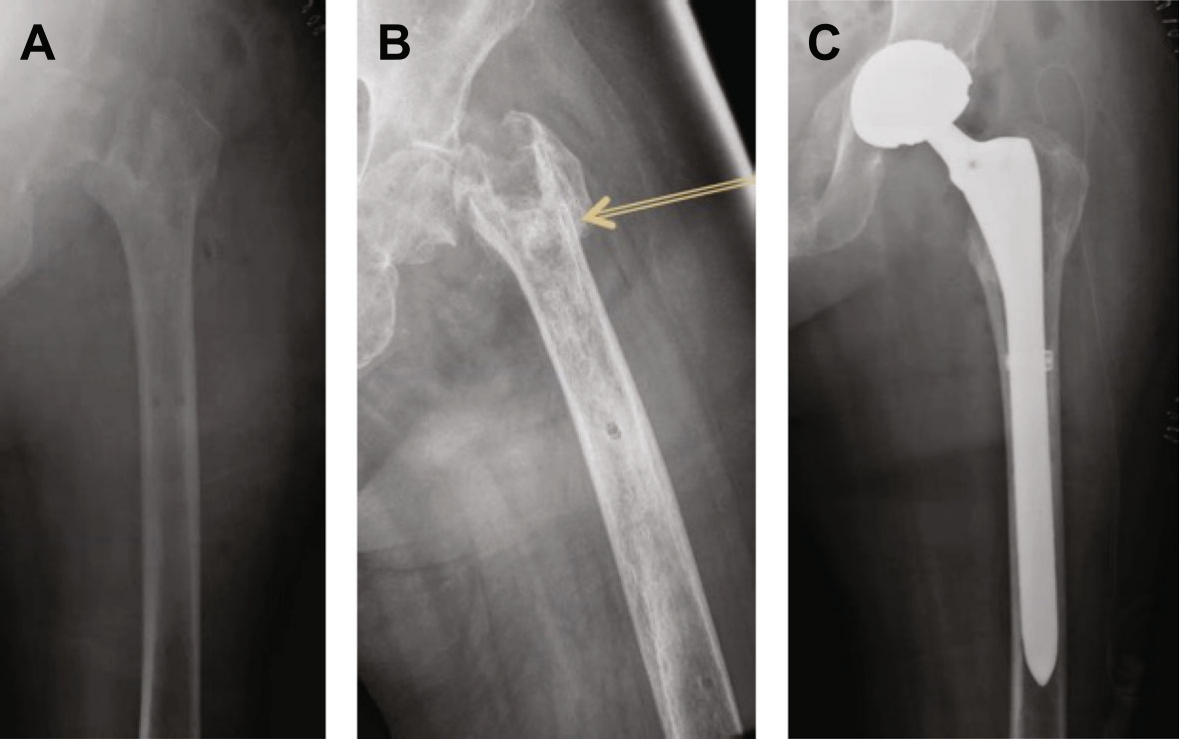
Post-operative radiographs of the 2-stage THA procedure in patient #2. **a** Post-operative radiograph for Stage 1 of THA. The fixation hardware was removed and the void was filled with V-CBA to rebuild the bone stock. **b** Radiograph 2 months after placing V-CBA showed some consolidation of the bone graft is evident (yellow arrow). **c** Post-operative radiograph for Stage 2 of THA. The second procedure was performed 3 months after V-CBA was implanted

Patient consent was obtained for publication of this case. A 64-year-old female presented with severe pain in her hip. She had a history of pelvic fracture and had fallen again resulting in placement of a femoral nail in January 2018 (Fig. 3a). The patient had Type I diabetes mellitus, wore an insulin pump, and had severe osteoporosis. The patient reported worsening pain 7 and 8 months after the initial surgery, and radiographs showed the hardware had shifted (Fig. 3b, c). By 11 months, the patient reported unbearable pain and her HHS was 17. Radiographs showed that the helical blade had perforated her femoral head and was in her pelvis and acetabulum (Fig. 3d). At this time, the hardware system was removed and 10 cc of V-CBA was implanted in Stage 1 of the THA (Fig. 4a). Radiographs taken 2 months after Stage 1 show some bone consolidation in the area where V-CBA was implanted (Fig. 4b). At this appointment, the patient’s Hgb A1c was 7.2, which was slightly elevated. Three months after Stage 1, the second stage of the THA was performed with placement of the total hip implant (Fig. 4c). A posterior approach was taken, and the femoral head and neck were removed. The acetabulum was reamed and trialed for fit. An excellent press-fit was obtained with a Biomet M2A Magnum mono block acetabular shell 48-mm outer diameter with a 42-mm inner diameter (Zimmer Biomet, Warsaw, IN). A small portion of the V-CBA was removed from the femoral canal and sent to the manufacturer for histological analysis. The femoral canal was reamed up to 15 mm and broached the femur with a size 15 stem. Through trialing, the leg length and offset were recreated, and the hip was stable. A Biomet Arcos one piece revision stem, 210-mm length, 15-mm diameter was implanted with an impacted Biomet Vitamin E Poly Dual Mobility head 42-mm bearing size with an inner Biomet Biolox Delta 28-mm Ceramic head and a neutral neck titanium alloy taper (Zimmer Biomet, Warsaw, IN). Due to her extreme osteoporosis, the treating surgeon felt it would be best to use a revision type stem to increase the surface area for porous attachment of bone to the implant. There were no peri-prosthetic fractures after implantation of the revision femoral stem; however, a prophylactic Kinamed cable was placed to prevent a future fracture. After discharge, the patient did not have any complications and was not readmitted.

### Histology

Formalin-fixed explant samples from two different patients were decalcified using bone decalcifier (American MasterTech #DCECDLT) for 7–8 h following the manufacturer’s instructions. The specimens were considered decalcified when they could be compressed. The samples were immersed in 10% buffered formalin until the DCI processing (dehydration, clearing, and paraffin infiltration) using Leica Automatic Tissue Processor TP1020. Paraffin embedded tissue samples were cut to 5–8-μm thickness on the largest surface of the samples. The routine hematoxylin and eosin (H&E) staining using Gill 2 hematoxylin solution (ready to use; Richard Allan Scientific #7231) and eosin Y alcoholic solution (ready to use, Richard Allan Scientific #7111) was performed per LifeNet Health protocol TM 40-014 [10]. Masson’s trichrome staining (American MasterTech, Catalog #KTMTR) was performed according to the manufacturer’s protocol. Histological slides were examined using a Zeiss microscope Axio Observer Z1 with objectives 10× and 20×, and images were captured by using a Zeiss camera AxioCam MRc and software Zen 2.3 pro.

## Results

### Case #1 results

At 3 months post-operative, the patient had not experienced any hospital readmissions, infections, dislocations, or fractures. His 3-month post-operative HHS had risen to 91.

**Fig. 5:**
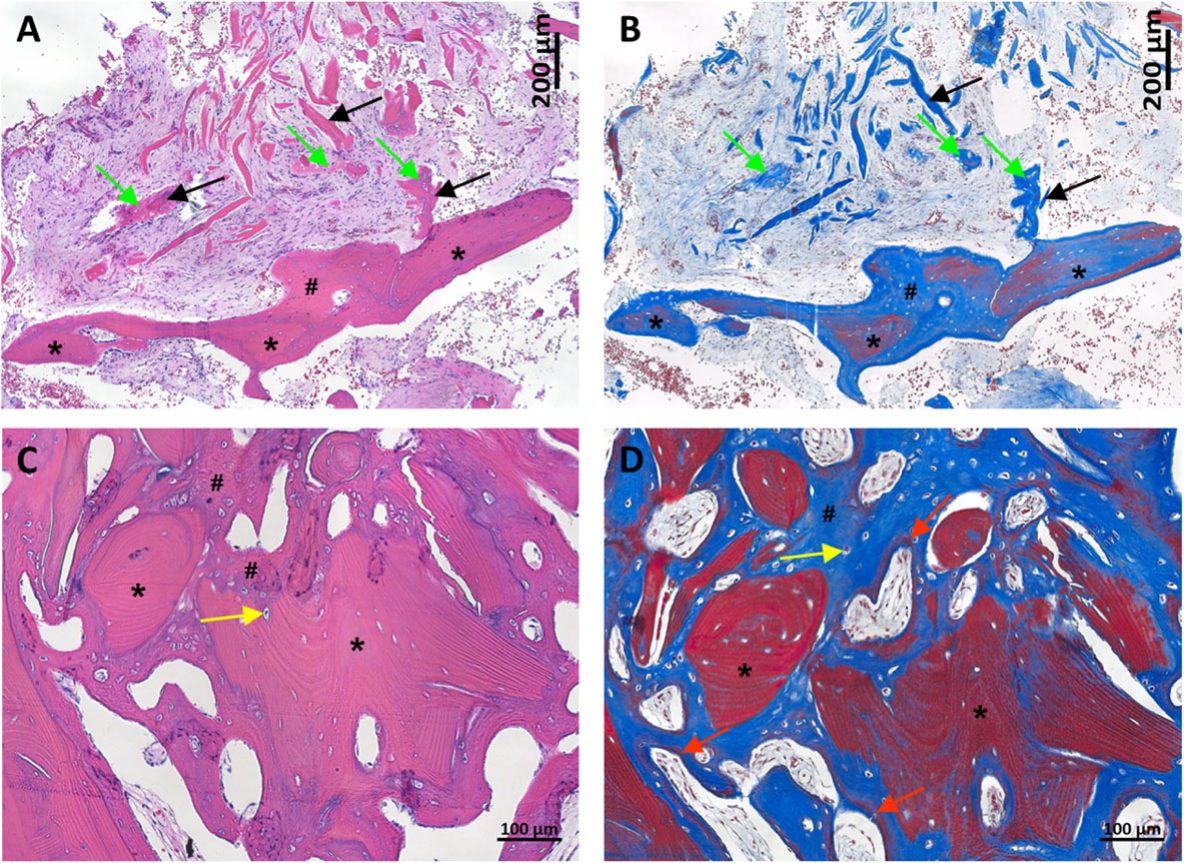
New bone formation 7 weeks after implantation of V-CBA into a femoral shaft. The femoral explant taken at 7 weeks following V-CBA implantation was fixed and sectioned (**a**, **b** and **c**, **d**), and then histologically stained with H&E (panels **a** and **c**) or Masson’s trichrome (panels **b** and **d**). The images included implanted V-CBA bone chips (*) and demineralized bone fibers (black arrows). Newly formed, pre-mineralized bone matrix was observed surrounding the implanted bone chips (#), as well as between the demineralized bone fibers (green arrows). Note the zone of new mineralization forming within the newly formed bone (red arrows, panel **d**). Yellow arrows indicate bone cells within lacunae. New bone fully covers the surfaces of implanted cortico-cancellous bone chips. The new bone matrix formed an interconnected structure, connecting implanted bone chips and fibers. Merged multiple 10× images (**a** and **b**) and 10× images (**c** and **d**)

**Fig. 6:**
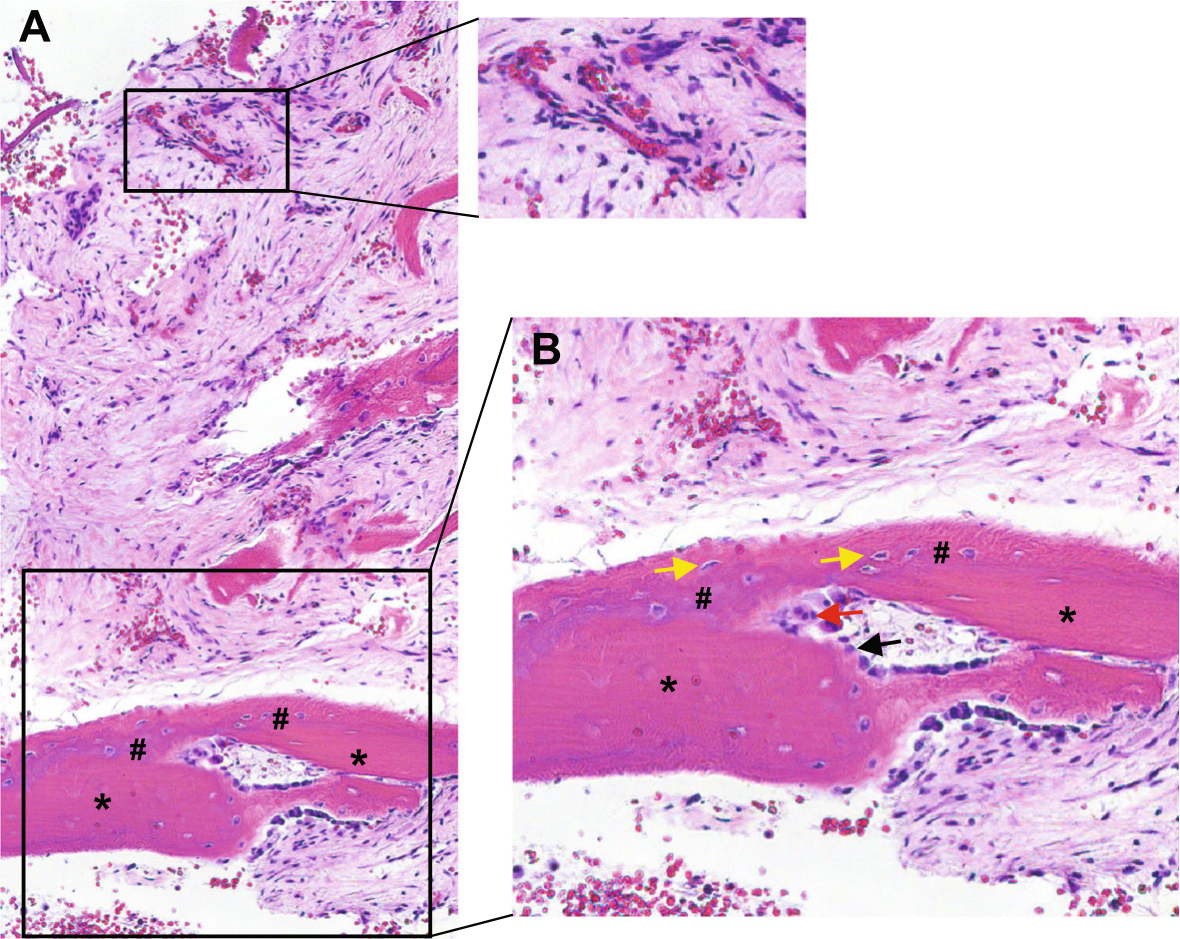
H&E stained section of the femoral explant showing new bone formation at 7 weeks. Panel **a** shows implanted V-CBA fibers with new bone formation between the fibers as well as neovascularization (top rectangle, expanded for better viewing). The bottom rectangle shows V-CBA bone chips (*) with new bone formation (#). Panel **b** shows the magnified area within the lower rectangle in panel **a**. Within the newly formed bone, osteocytes (yellow arrows) occupy the spaces within the lacunae. Osteoblasts (black arrow), with their characteristic cuboidal shape, can be seen along the edges of the newly formed bone depositing bone matrix. Osteoclasts (red arrow) with large multinucleated morphology are leading the new bone formation path for osteoblasts. Merged multiple 10× images (**a**) and 10× image (**b**)

The explant was examined histologically and showed substantial new bone formation at 7 weeks following V-CBA implantation (Fig. 5 and 6). Masson’s trichrome staining demonstrated new bone (#) surrounded the implanted V-CBA bone chips, which are stained red with striations (*). Likewise, in the areas between the implanted demineralized bone fibers (black arrows), new bone was observed (green arrows). Non-striated red staining along the edges of the newly formed bone (red arrows, panel D, Fig. 5) demonstrate areas of new mineralization. Osteocytes occupy the spaces within lacunae (yellow arrows). Osteoclasts with the large multinucleated morphology, and osteoblasts, with their characteristic cuboidal shape (red and black arrows, respectively, in Fig. 6), surround the edges of the newly formed bone. These results suggest that V-CBA-derived cells, such as osteoblasts, directly participate in bone formation via an intramembranous ossification pathway. The implanted V-CBA and demineralized fibers appear to be actively remodeling resulting in the formation of new bone matrix around them. The presence of osteocytes in the lacunae of the bone chips along with osteoblasts lining the new bone is consistent with osteoinductive and osteogenic properties of the implant.

### Case #2 results

Two months after the THA was performed, the patient’s HHS had improved to 77.

**Fig. 7:**
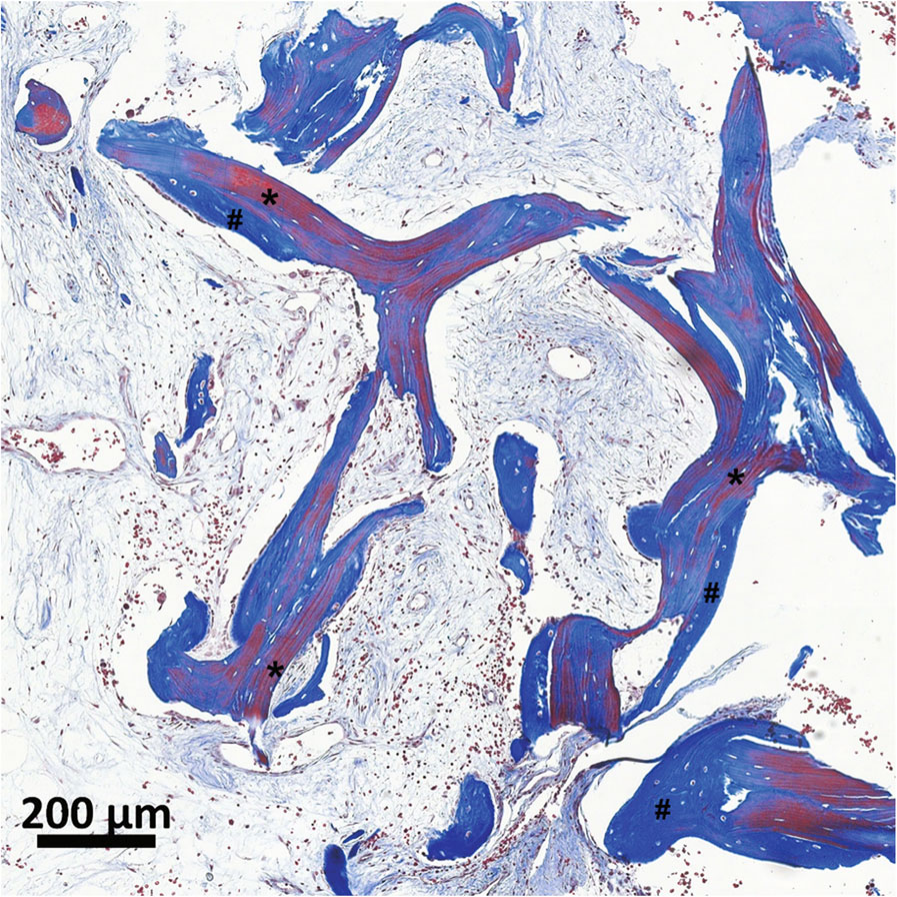
New bone formation 3 months after V-CBA implantation. Masson’s trichrome staining of a section of the femoral explant taken 3 months after V-CBA implantation shows new bone formation (#) where the V-CBA bone chips (*) were implanted. The surrounding tissue is also in healthy remodeling phase supported by neovascularization and without signs of rejection. Merged 10× images

**Fig. 8:**
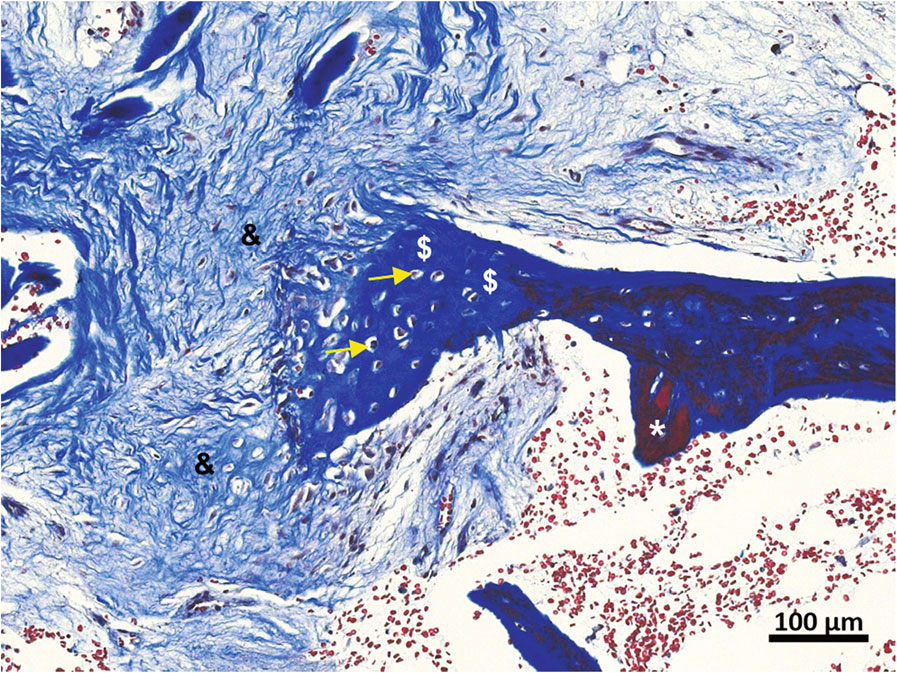
Progression of new bone formation at 3 months post-implantation of V-CBA. A trichrome-stained section of the femoral explant taken 3 months after V-CBA implantation demonstrates the progression of new bone formation. Collagen fibers (&) are seen gradually organizing to consolidate into pre-mineralized bone matrix ($) and encapsulating osteocytes within lacunae (yellow arrows). Magnification 10×

This explant was examined histologically by H&E and Masson’s trichrome staining for new bone formation (Figs. 7 and 8). While less extensive than in the previous case, new bone (#) was evident surrounding the V-CBA bone chips (*; Fig. 7). Notably, the stained section presented in Fig. 8 depicts the progression of gradual collagen deposition during bone formation. The fibers have become integrated with each other, forming an organized solid bony matrix, indicative of being further progressed in the bone formation process*.* Within this osteoid, the osteoblasts have become encapsulated within the lacunae of the bone to become osteocytes.

## Discussion

It is difficult to assess short-term *in situ* performance of implanted bone graft materials because the implanted graft usually remains undisturbed for months or years unless a revision surgery is needed. However, in the two-stage approach to THA used in these cases, there was a unique opportunity to examine graft-driven bone formation via histologic analysis of explants.

The clinical outcomes and histology from these cases demonstrated that V-CBA is capable of rapidly eliciting new bone formation in distinctly different patients. In the first case, the patient was a 48-year-old male with severe hip osteoarthritis who was otherwise healthy. The radiographs showed good graft consolidation at 7 weeks after V-CBA had been implanted. The literature suggests that active new bone formation may occur at a rate of 1–2 μm/day [11]. As shown here for V-CBA implants (Fig. 5), several areas show cumulative new bone formation of over 100 μm after only 7 weeks. This suggests that new bone formation started directly after implantation and continued at a rapid pace. This early mineralization and absence of chondrocytes suggests that V-CBA forms bone mostly via the more direct intramembranous pathway. In the second case, the patient was a 64-year-old woman with Type I diabetes, osteoporosis, and a history of pelvic fracture. This explant was procured after V-CBA had been implanted for 12 weeks. Diabetes, age, and gender can make bone formation and healing more challenging [12, 13]. This is evident by the difference in histology between the two patients: although active and extensive new bone formation is also seen in the second patient, it appears to be at a less mature stage at 12 weeks compared to the first patient after only 7 weeks. However, even in this difficult-to-heal patient, use of V-CBA resulted in enough osteoid formation to help support the new implant. In both patients, the explanted V-CBA showed neovascularization and was without any signs of immune rejection.

Although autologous bone is still considered by some to be the gold standard for bone grafting, it is important to acknowledge that its quality is dependent upon the health of the patient, as well as how it is procured [14]. With an aging population, many patients have comorbidities or take medications that may result in poor bone quality [15, 16]. Even for healthy patients, an additional concern is that autologous bone procurement opens a second site that risks infection and potentially prolongs overall healing [17]. As surgical site infections are common, an unnecessary second surgery site represents not only a health risk to the patient, but also a financial risk to the hospital [17, 18]. An allograft that can mirror the desirable properties of autograft while avoiding the potential risks is a valuable option. Like autograft, V-CBA has all three properties necessary for bone formation: osteoconductivity, osteoinductivity, and osteogenicity. While not generalizable, the difference in new bone formation between the distinct patients presented here is suggestive of the strong role comorbidities play in bone healing. However, it also demonstrates that V-CBA may facilitate new bone formation even in challenging patients. Patients, surgeons, and hospitals can all benefit from having an advanced CBA that eliminates the need for autologous bone grafting.

## Conclusions

Although limited to two patients, this is the first publication showing the efficacy of V-CBA cellular bone allograft via histological results in human subjects. V-CBA elicited new bone formation in two distinctly different patients, one of whom had multiple comorbidities that put her at high risk for delayed bone formation. These cases suggest that V-CBA is a suitable replacement for autograft bone in two stage THA.

## Data Availability

All data generated or analyzed during this study are included in this published article.

## Abbreviations

CBA: Cellular bone allograft
H&E: Haemotoxylin and eosin
HHS: Harris Hip Score
THA: Total hip arthroplasty
V-CBA: ViviGen formable cellular bone allograft
